# Association of intercellular adhesion molecule 1 (ICAM-1) (rs5498) genetic polymorphism and dengue in Sabah East Malaysia population

**DOI:** 10.1007/s11033-025-11256-x

**Published:** 2025-11-28

**Authors:** Rhanye Mac Guad, Yin Nwe Aung, Andrew W. Taylor-Robinson, Carlos Silvester, Maw Shin Sim, Lee Heng Gee, Wu Yuan Seng, Shamala Devi Sekaran, Nornazirah Azizan

**Affiliations:** 1 Department of Biomedical Sciences, Faculty of Medicine and Health Science, Universiti Malaysia Sabah, Kota Kinabalu, Sabah, Malaysia; 2 Faculty of Medicine and Health Sciences, UCSI University, Springhill Campus, Port Dickson, Negeri Sembilan Malaysia; 3 College of Health Sciences, VinUniversity, Gia Lam district, Hanoi, Vietnam; 4 Center for Global Health, Perelman School of Medicine, University of Pennsylvania, Philadelphia, PA USA; 5 Department of Pharmaceutical Life Sciences, Faculty of Pharmacy, Universiti Malaya, Kuala Lumpur, Malaysia; 6 Precision Medicine and Omics Centre (PrOmiC), Faculty of Pharmacy, Universiti Malaya, Kuala Lumpur, Malaysia; 7 Department of Medicine, Queen Elizabeth Hospital , Kota Kinabalu, Sabah Malaysia; 8 Department of Biomedical Sciences, Sir Jeffrey Cheah Sunway Medical School, Faculty of Medical and Life Sciences, Sunway University, Subang Jaya, Malaysia; 9 Sunway Microbiome Centre, Faculty of Medical and Life Sciences, Sunway University, Subang Jaya, Malaysia; 10 Department of Pathology and Microbiology, Faculty of Medicine and Health Science, Universiti Malaysia Sabah, Kota Kinabalu, Sabah, Malaysia

**Keywords:** Dengue, ICAM-1, Genetic polymorphism, Sabahan population, Susceptibility

## Abstract

**Background:**

Dengue remains a significant public health challenge in Malaysia, particularly in Sabah where indigenous populations such as the Kadazan-Dusun and Bajau are highly affected. Host genetic factors, including polymorphisms in immune-related genes, may influence individual susceptibility to dengue infection. This study investigates the association between Intercellular Adhesion Molecule-1 (ICAM-1) rs5498 (K469E) polymorphism and dengue infection in the Sabahan population, with a focus on ethnic and gender stratification.

**Methods:**

A total of 83 participants were recruited, comprising 55 laboratory-confirmed dengue cases and 28 healthy controls, stratified by ethnicity (Kadazan-Dusun, Bajau) and gender. Genotyping of ICAM-1 rs5498 was performed using a Fluidigm Genotyping Array. Genotype and allele frequencies were compared using chi-square tests. Odds ratios (ORs) with 95% confidence intervals (CIs) were calculated to evaluate associations under various genetic models.

**Results:**

The GG genotype and G allele were significantly more frequent among dengue cases compared with controls (GG: 56.4% vs. 17.9%; G allele: 64.6% vs. 35.7%; *p* < 0.01). Subgroup analysis revealed significant associations in both Kadazan-Dusun (*p* = 0.017) and Bajau (*p* = 0.015) participants, with the A allele conferring a protective effect (overall OR = 0.31, 95% CI: 0.16–0.60). Gender-stratified analysis showed stronger associations in females, where the GG genotype (68.2% vs. 9.1%) and G allele (70.5% vs. 22.7%) were significantly enriched among cases (*p* ≤ 0.003). Logistic regression under a recessive model confirmed the protective role of the A allele (adjusted OR = 0.19, 95% CI: 0.06–0.59).

**Conclusion:**

The ICAM-1 rs5498 GG genotype and G allele are significantly associated with increased susceptibility to dengue infection in the Sabahan population, particularly among females. These findings highlight the potential role of ICAM-1 as a genetic biomarker for dengue risk and underscore the importance of considering ethnic and gender differences in genetic epidemiology studies of infectious diseases.

## Introduction

Sabah, located on Borneo Island, is home to a diverse multi-ethnic population [1]. Among these, the Kadazan-Dusun and Bajau represent the two largest indigenous groups, each with distinct cultural practices and genetic backgrounds. The Kadazan-Dusun, traditionally inland agriculturalists, and the Bajau, historically maritime seafarers [2], reflect distinct lifestyle and environmental exposures that may influence host–vector interactions and immune function [3, 4]. Both groups share Austronesian ancestry but have diverged genetically due to separate migration histories and limited intermarriage [5], leading to observable differences in allele frequencies of immune-related genes in prior studies.

Between 2013 and 2018, Sabah experienced a substantial increase in dengue cases, with reported numbers escalating from 724 to 3,423 cases [6]. This surge was accompanied by a notable rise in mortality, especially in the eastern districts such as Tawau, Semporna, Lahad Datu, and Sandakan (Bajau majority) as well as Kota Belud, Tuaran, and Penampang (Kadazan-Dusun majority) [7].

The severity of dengue infection varies among ethnicities, and host genetic factors have been implicated in influencing disease outcomes [8].Despite the high burden of dengue in Sabah, there is a paucity of data on the genetic factors contributing to disease susceptibility and severity within this population. Given the ethnic diversity and unique genetic makeup of the Sabahan population, studies addressing host genetic variation may provide valuable insights into disease pathogenesis and inform targeted control strategies.

One candidate gene of particular interest is *Intercellular Adhesion Molecule 1* (ICAM-1), a cell surface glycoprotein expressed on endothelial cells and immune cells, that plays a pivotal role in leukocyte adhesion and transmigration during inflammatory responses [9]. The ICAM-1 gene harbours a functional single nucleotide polymorphism (SNP), rs5498 (also known as K469E), which results in a lysine to glutamic acid substitution at codon 469 [10]. This polymorphism has been associated with various inflammatory and infectious diseases, including dengue [11, 12]. Although only limited studies have directly evaluated this polymorphism [13], broader evidence supports the involvement of ICAM-1 in dengue pathogenesis. Increased ICAM-1 expression and elevated soluble ICAM-1 levels have been associated with endothelial activation, enhanced leukocyte adhesion, and greater vascular permeability in severe dengue cases [14–16]. These pathways provide biological plausibility that genetic variation at rs5498 may modulate susceptibility to dengue infection.

In light of the substantial dengue burden in Sabah and the paucity of genetic studies in this region, investigating the ICAM-1 rs5498 polymorphism offers an important opportunity to better understand host susceptibility factors. This study aims to evaluate the association between the ICAM-1 rs5498 polymorphism and susceptibility to dengue infection among individuals in Sabah. Understanding this relationship may contribute to the development of genetic markers for predicting disease risk and severity, ultimately aiding in the implementation of personalized medical approaches and effective disease management strategies in the region.

## Materials and methods

### Study design

The study subjects were recruited either retrospectively from the database available at Jabatan Kesihatan Negeri Sabah (JKNS) or prospectively from public hospitals in Sabah. The study subjects were divided into case and control groups. Cases group consist of patients with dengue while control group consist of healthy adult and no history of dengue infection. All dengue cases were confirmed by laboratory testing at Sabah public hospitals, including NS1 antigen detection, RT-PCR, and/or dengue-specific IgM/IgG serology. Adults aged above 18 years old, diagnosed with dengue and consented to participate were included in this study. Subjects with major medical illness (including chronic cardiovascular disease, chronic kidney disease, malignancies, autoimmune diseases, and immunosuppressive conditions), mixed with non-indigenous ethnicity, or who declined testing procedures were excluded.

### Sample size calculation

Sample size was calculated using Quanto version 1.2.4 [17] for case-control studies, considering the expected frequency of the rs5498 polymorphism in the population, an anticipated odds ratio, a significance level of 0.05, and a power of 80%.

### Data and sample collection

The structured questionnaire was pretested among a pilot group of participants (*n* = 20) to assess clarity and cultural appropriateness. Feedback from the pretest was used to refine the wording of several items, ensuring that the final version was easily understood across both Kadazan-Dusun and Bajau respondents. The final, structured questionnaires were administered to collect information on age, gender, ethnicity, residential area, and potential exposure to dengue vectors. Regarding biological samples, three millilitres of blood from the study subjects were collected in ethylenediaminetetraacetic acid (EDTA) tube according to a standard method. DNA was extracted from leukocytes by using PrimePrep™ Genomic DNA Extraction Kit (GeNet Bio, Korea). The concentration of extracted DNA was measured by using NanoDrop™ Spectrophotometer (Thermo Scientific™, USA). DNA samples were stored in refrigerator at −20 °C.

### DNA genotyping

Genotyping of the ICAM-1 SNP (rs5498) was performed using the Fluidigm SNP Type™ Genotyping System (Fluidigm Corporation, South San Francisco, CA) [18], which leverages microfluidic technology for high-throughput and precise genetic analysis. Specific Target Amplification (STA) was employed to enhance the detection sensitivity of the target SNPs. The genotyping assays were conducted on the Fluidigm 96.96 Dynamic Array™ Integrated Fluidic Circuit (IFC), which enables the simultaneous analysis of 96 samples against 96 assays, resulting in 9,216 individual reactions per run. The IFC was loaded with the prepared assay and sample mixtures using the IFC Controller MX, following the manufacturer’s instructions. Thermal cycling and fluorescence detection were performed on the BioMark™ HD System (Fluidigm, USA), which provides real-time PCR capabilities and high-resolution imaging for precise genotyping. Data analysis was carried out using the Fluidigm SNP Genotyping Analysis software, which automatically interprets fluorescence signals to determine genotype calls.

### Hardy–Weinberg equilibrium (HWE) testing

The genotype distributions for each SNP were assessed for compliance with HWE using the chi-square test. Upon analysis, both the case and control groups exhibited significant deviation from HWE (*p* < 0.001), indicating a statistically significant departure from equilibrium for this SNP. To further explore this observation, stratified testing by age was conducted. It was found that control participants aged ≥ 24 years were not in HWE, whereas those < 24 years conformed to HWE expectations. To minimize bias due to population substructure or genotyping errors, the final genetic association analysis was restricted to the subgroup of participants younger than 24 years, where the HWE assumption was met. Consequently, a total of 83 participants were included in the final analysis (compared with the original 382 participants initially genotyped).

### Statistical analysis

Mean (± standard deviation or SD) for continuous data was utilised to analyse and report the demographic features of the study participants, and a t-test was applied to compare the dengue cases and control. When necessary, the chi-square (X^2^) test and Fisher exact were used for intergroup statistical analysis of categorical variables. By using categorical variables, the genotype frequencies of various genotypes will be compared between dengue cases and controls. The dengue cases and control were compared on an overall basis, and subgroup comparisons by race and gender were also conducted. A chi-square test and, if necessary, a Fisher exact test was also conducted when the sample size was too small, and the presence of group differences was determined by their p-value. A p-value of less than 0.05 is considered to be a statistically significant difference. To assess the allelic frequencies of dengue cases and control, both heterozygous and homozygous genes were examined. These include odds ratio and the allele frequencies of the subgroups of race and gender. The odds ratio of genotypes among dengue cases relative to the controls was determined using logistic regression. The Bonferroni correction is used to alter the p-value due to multiple observations for the significant cut-off value. Statistical analysis was performed using strata 6 software.

## Results

### Demographic characteristics of study participants

A total of 83 participants were included, comprising 60 Kadazan-Dusun and 23 Bajau individuals. Among the Kadazan-Dusun, 36 were cases and 24 were controls, with a mean age of 18.0 years (SD = 4.39) for cases and 18.8 years (SD = 3.64) for controls. In the Bajau group, there were 19 cases and 4 controls, with mean ages of 17.8 years (SD = 4.45) and 21.0 years (SD = 1.82), respectively. When combined, the overall mean age was 17.9 years (SD = 4.37) for cases and 19.1 years (SD = 3.51) for controls. Gender distribution was comparable across groups. Among the Kadazan-Dusun, males accounted for 54.3% of cases and 45.7% of controls, while females comprised 68.0% of cases and 32.0% of controls. In the Bajau group, 93.3% of cases were male compared to 6.7% of controls, whereas females represented 62.5% of cases and 37.5% of controls. Overall, males constituted 66.0% of cases and 34.0% of controls, while females made up 66.7% of cases and 33.3% of controls. No statistically significant differences were observed between cases and controls for either age or gender distribution across both ethnic groups (Table 1).

**Table 1:** Demographic characteristics of the study participants

Characteristic	Kadazan-Dusun (*n* = 60)			Bajau (*n* = 23)			Total (*n* = 83)		
	Case (*n* = 36)	Control (*n* = 24)	*p*	Case (*n* = 19)	Control (*n* = 4)	*p*	Case (*n* = 55)	Control (*n* = 24)	*p*
Age, mean (SD), year	18.00 (4.39)	18.75 (3.64)	0.49	17.79 (4.45)	21 (1.82)	0.18	17.93 (4.37)	19.07 (3.51)	< 0.23
Gender									
Male, N (%)	19 (54.29)	16 (45.71)	0.285	14 (93.33)	1 (6.67)	0.103*	33 (66.00)	17 (34.00)	0.950
Female, N (%)	17 (68.00)	8 (32.00)		5 (62.50)	3 (37.50)		22 (66.67)	11 (33.33)	

Table 2 presents the genotype and allelic frequencies of the ICAM-1 SNP (rs5498) among dengue cases and controls, stratified by ethnicity (Kadazan-Dusun and Bajau). Genotype distributions are categorized as AA, AG, and GG, while allele frequencies are reported as A and G. Among Kadazan-Dusun participants, cases showed higher frequencies of the GG genotype (50.0%) compared with controls (20.8%), whereas the AA genotype was less common among cases (30.6% vs. 45.8%). Although the genotype distribution did not reach statistical significance (*p* = 0.073), the G allele was significantly more frequent in cases (59.7%) than in controls (37.5%) (*p* = 0.017), conferring a reduced odds of disease for the A allele (OR = 0.41, 95% CI: 0.19–0.86). In the Bajau group, the GG genotype predominated among cases (68.4%) but was absent in controls, with significant differences observed in both genotype (*p* = 0.030) and allele distributions (*p* = 0.015). The G allele was markedly more frequent in cases (73.7%) compared with controls (25.0%), corresponding to a protective effect of the A allele (OR = 0.12, 95% CI: 0.02–0.69). When both ethnicities were combined, genotype frequencies showed significant variation (*p* = 0.003), with the GG genotype overrepresented among cases (56.4%) relative to controls (17.9%). Similarly, the G allele was significantly more common in cases (64.6%) than in controls (35.7%) (*p* = 0.001). Overall, carriage of the A allele was associated with reduced dengue risk (OR = 0.31, 95% CI: 0.16–0.60).

**Table 2:** Genotype and allelic frequencies of *ICAM-1* SNP (rs 5498) based on ethnicity

Ethnicity	Participant	Genotype Frequency, n (%)				Allelic Frequency, n (%)			OR (95% CI)
		AA	AG	GG	*p*	A	G	*p*	
Kadazan-Dusun	Case	11 (30.56)	7 (19.44)	18 (50.00)	0.073	29 (40.28)	43 (59.72)	0.017	0.405 (0.191–0.857)
	Control	11 (45.83)	8 (33.33)	5 (20.83)		30 (62.50)	18 (37.50)		
Bajau	Case	4 (21.05)	2 (10.53)	13 (68.42)	0.030*	10 (26.32)	28 (73.68)	0.015*	0.119 (0.021–0.689)
	Control	2 (50.00)	2 (50.00)	0 (0.00)		6 (75.00)	2 (25.00)		
Total	Case	15 (27.27)	9 (16.36)	31 (56.36)	0.003	39 (35.45)	71 (64.55)	0.001	0.305 (0.156–0.597)
	Control	13 (46.43)	10 (35.71)	5 (17.86)		36 (64.29)	20 (35.72)		

Note: Boldface shows a significant difference, *p* < 0.05; CI, confidence interval; OR, Odds Ratio *fisher exact test is used

Table 3 presents the genotype and allelic frequencies of the ICAM-1 SNP (rs5498) stratified by gender among dengue cases and controls. The distribution of ICAM-1 rs5498 genotypes varied according to gender. Among male participants, genotype frequencies did not differ significantly between cases and controls (*p* = 0.246). However, the G allele was more frequent in cases (60.6%) than in controls (44.1%), though this difference was not statistically significant (*p* = 0.116). In contrast, significant differences were observed among female participants. The GG genotype was markedly more common in cases (68.2%) than in controls (9.1%), while the AA genotype was substantially lower among cases (27.3% vs. 63.6%) (*p* = 0.003). At the allelic level, the G allele was significantly overrepresented in cases (70.5%) compared to controls (22.7%) (*p* = 0.001), with carriage of the A allele associated with reduced dengue risk (OR = 0.12, 95% CI: 0.04–0.41). When both genders were combined, significant differences persisted in both genotype (*p* = 0.003) and allele frequencies (*p* = 0.001), with the G allele more common among cases (64.6%) than controls (35.7%). Overall, the presence of the A allele was protective (OR = 0.31, 95% CI: 0.16–0.60).

**Table 3:** Genotype and allelic frequencies of *ICAM-1* SNP (rs 5498) based on gender

Gender	Participant	Genotype Frequency, n (%)				Allelic Frequency, n (%)			OR (CI 95%)
		AA	AG	GG	*p*	A	G	*p*	
Male	Case	9 (27.27)	8 (24.24)	16 (48.48)	0.246*	26 (39.39)	40 (60.61)	0.116	0.513 (0.222–1.186)
	Control	6 (35.29)	7 (41.18)	4 (25.53)		19 (55.88)	15 (44.12)		
Female	Case	6 (27.27)	1 (4.55)	15 (68.18)	0.003*	13 (29.55)	31 (70.45)	0.001	0.123 (0.037–0.405)
	Control	7 (63.64)	3 (27.27)	1 (9.09)		17 (77.27)	5 (22.73)		
Total	Case	15 (27.27)	9 (16.36)	31 (56.36)	0.003	39 (35.45)	71 (64.55)	0.001	0.305 (0.156–0.597)
	Control	13 (46.43)	10 (35.71)	5 (17.86)		36 (64.29)	20 (35.72)		

Note: Boldface shows a significant difference, *p* < 0.05; CI, confidence interval; OR, Odds Ratio. * Fisher exact test is used. L

Table 4 presents the odds ratios (OR) and 95% confidence intervals (CI) for the association between different genotypes of the ICAM-1 SNP (rs5498) and dengue infection under various genetic models. The association between ICAM-1 rs5498 genotypes and dengue risk was further evaluated using logistic regression under recessive and dominant models. In the recessive model (AG + AA vs. GG), carriers of the A allele (AG + AA) had a significantly reduced risk of dengue infection compared with the GG genotype. The univariate analysis yielded an odds ratio (OR) of 0.17 (95% CI: 0.06–0.51, *p* = 0.002), which remained significant after adjustment for gender, ethnicity, and age (adjusted OR = 0.19, 95% CI: 0.06–0.59, *p* = 0.004). In the dominant model (AG + GG vs. AA), carriers of the G allele showed an increased risk of dengue compared with the AA genotype, although this association did not reach statistical significance (univariate OR = 2.31, 95% CI: 0.89–5.98, *p* = 0.084; adjusted OR = 2.32, 95% CI: 0.86–6.29, *p* = 0.097). These findings suggest that the recessive model provides stronger evidence for the protective effect of the A allele, while the dominant model showed a non-significant trend toward risk associated with the G allele.

**Table 4:** Logistic regression analysis of genotypes under recessive and dominant models

Genetic Model	Genotype	OR (Univariate) [95% CI]	*p*	OR (Multivariate)* [95% CI]	*p*
**Recessive**	GG (Ref)	1.00	–	1.00	–
	AG + AA	0.17 (0.06–0.51)	0.002	0.19 (0.06–0.59)	0.004
**Dominant**	AA (Ref)	1.00	–	1.00	–
	AG + GG	2.31 (0.89–5.98)	0.084	2.32 (0.86–6.29)	0.097

Note: Boldface shows a significant difference, *p* < 0.05; OR = Odds Ratio; CI = Confidence Interval; Ref = reference group. Multivariate analysis adjusted for gender, race, and age

## Discussion

This study investigated the association between ICAM-1 SNP rs5498 and susceptibility to dengue infection among the Kadazan-Dusun and Bajau ethnic groups in Sabah, East Malaysia. Our results provide compelling evidence that the GG genotype and the G allele of rs5498 are significantly associated with an increased risk of dengue infection, particularly among females and the Kadazan-Dusun ethnic.

We observed a consistent overrepresentation of the GG genotype and the G allele among cases compared with controls across both Kadazan-Dusun and Bajau populations. In Kadazan-Dusun participants, the G allele was significantly associated with increased dengue risk, with the A allele conferring a protective effect. Similarly, Bajau participants, particularly females, showed a strong enrichment of the G allele among cases. Similarly, a study conducted among Indian populations [9] found that individuals homozygous GG had a higher risk of developing severe forms of dengue, such as dengue hemorrhagic fever (DHF) and dengue shock syndrome (DSS). This association was attributed to elevated mRNA expression levels of ICAM-1, suggesting that the GG genotype may enhance disease severity.

Logistic regression analyses supported these observations. The recessive model (AG + AA vs. GG) revealed a strong protective effect of the A allele, which remained significant after adjusting for confounders, suggesting a biologically relevant role of this variant in dengue pathogenesis. Conversely, the dominant model indicated a trend toward increased risk with G allele carriage, though this did not reach statistical significance. Collectively, these results indicate that the protective effect of the A allele is best explained under a recessive framework, whereas the G allele is more consistently associated with increased risk.

Furthermore, it has demonstrated that severe dengue virus infections induce the expression of macrophage migration inhibitory factor (MIF) [19], which in turn stimulates monocytes and endothelial cells to express higher levels of ICAM-1. This upregulation of ICAM-1 is implicated in the pathogenesis of severe dengue, supporting the notion that genetic variations leading to increased ICAM-1 expression, such as the rs5498 G allele, may contribute to disease severity. The consistent observation across different populations and disease contexts underscores the potential of rs5498 as a genetic marker for disease susceptibility and severity. To advance understanding, future studies should investigate genotype–phenotype relationships by assessing ICAM-1 expression levels, circulating sICAM-1 concentrations, and functional assays. Such approaches will help clarify how rs5498 influences leukocyte adhesion, endothelial interactions [20], and ultimately susceptibility to dengue infection, thereby strengthening its relevance for clinical risk assessment.

Notably, gender-stratified analysis revealed stronger associations among females. Female cases exhibited significantly higher frequencies of the GG genotype and G allele than controls, implying that females may be more genetically predisposed to dengue infection due to this polymorphism. These findings are further supported by the ethnicity-gender subgroup analysis, plausibly influenced by a combination of biological, immunological, and hormonal factors. Females generally exhibit stronger innate and adaptive immune responses compared to males [21], which, while beneficial in many infectious contexts, can also predispose them to heightened inflammatory reactions and more severe disease outcomes. Estrogen and other sex hormones are known to modulate immune system activity [22], potentially enhancing the expression of receptors or signaling pathways exploited by the dengue virus for cellular entry and replication. Additionally, gender-based differences in cytokine responses—particularly in the production of pro-inflammatory mediators [23]—may contribute to the exaggerated immune responses that characterize severe dengue manifestations. Finally, sociocultural factors, such as differences in exposure or health-seeking behaviors [24, 25] of the Kadazan-Dusun and Bajau ethnicities, may also play a minor role, though the biological basis is more consistently supported by current evidence. SNPs may further interact with these sex-specific immune mechanisms, making certain alleles or genotypes more impactful in females.

The ICAM-1 gene encodes a cell surface glycoprotein expressed on endothelial cells and immune cells, playing a pivotal role in leukocyte adhesion and transmigration during inflammatory responses [9]. The rs5498 polymorphism results in a K469E amino acid substitution, which may alter the molecule’s binding affinity or expression [26, 27], potentially affecting immune cell recruitment to infection sites. Our findings suggest that individuals with the GG (Glu/Glu) genotype may exhibit an altered immune response, rendering them more susceptible to severe dengue pathogenesis.

The study’s strength lies in its ethnically diverse and balanced case-control design, allowing meaningful stratified analysis by gender and ethnicity. However, limitations include the modest sample size in some subgroups (e.g., Bajau males), which may reduce statistical power. Additionally, potential confounders such as disease severity, secondary infection status, and environmental exposure were not fully accounted for. Finally, complete hematological and biochemical data (including platelet count, hematocrit, liver enzymes) were not consistently available for all cases.

Although this study demonstrates a significant association between the ICAM-1 rs5498 polymorphism and dengue susceptibility, the findings remain correlative in nature and do not establish a direct causal relationship. Functional validation was not performed, as the current study was originally designed as a genetic association analysis and did not include serum or RNA collection for downstream expression or protein quantification assays. Consequently, no direct evidence linking rs5498 genotypes to ICAM-1 mRNA or protein expression, or to serum soluble ICAM-1 (sICAM-1) concentrations, could be evaluated. The observed associations should therefore be interpreted cautiously as indicative rather than mechanistic. Future studies incorporating genotype-stratified analyses of ICAM-1 gene expression, sICAM-1 serum levels, and in vitro functional assays are warranted to substantiate the biological implications of this variant and to elucidate its precise role in dengue pathogenesis.

In conclusion, this study highlights a significant association between ICAM-1 rs5498 polymorphism and susceptibility to dengue infection, particularly among female and Kadazan-Dusun individuals. The GG genotype and G allele appear to confer increased risk, potentially due to their role in immune modulation. These findings warrant further investigation in larger cohorts and functional studies to elucidate the underlying mechanisms and explore the potential of ICAM-1 as a genetic marker for dengue risk stratification in endemic regions. It is also suggested that a combine host genetic profiling with viral serotyping to be conducted to explore potential serotype-specific effects of ICAM-1 polymorphisms on dengue susceptibility and severity for this population. Additionally, the absence of functional or expression-level validation (such as ICAM-1 mRNA, protein, or sICAM-1 measurement) limits mechanistic inference and should be addressed in future studies.

## Data Availability

No datasets were generated or analysed during the current study.

## References

[1] Hoh BP, Deng L, Xu S (2022) The peopling and migration history of the natives in Peninsular Malaysia and Borneo: a glimpse on the studies over the past 100 years. Front Genet 13:767018 35154269 10.3389/fgene.2022.767018 PMC8829068

[2] Peng TN, Li LS, Lian JCK (2022) Demographic and socioeconomic changes in Sabah. Universiti Malaysia Sabah, Kota Kinabalu

[3] Eleftherianos I, Zhang W, Tettamanti G, Daley L, Mohamed A, Stanley D (2024) Nutrition influences immunity: diet and host-parasite interactions. Insect Biochem Mol Biol 175:104210 39515668 10.1016/j.ibmb.2024.104210

[4] de Souza WM, Weaver SC (2024) Effects of climate change and human activities on vector-borne diseases. Nat Rev Microbiol 22(8):476–491 38486116 10.1038/s41579-024-01026-0

[5] Ali I, Abdullah MF (2024) Tracing the heritage and civilization of Austronesian seafarers across waters, seas, and oceans in the Archipelago of Southeast Asia. Applied History Journal of Merong Mahawangsa 2:41–55

[6] Murphy A, Rajahram GS, Jilip J, Maluda M, William T, Hu W et al (2020) Incidence and epidemiological features of dengue in Sabah, Malaysia. PLoS Negl Trop Dis 14(5):e0007504 32392222 10.1371/journal.pntd.0007504 PMC7241834

[7] Kaur N, Rahim SSSA, Jaimin JJ, Dony JJF, Khoon KT, Ahmed K (2020) The East Coast districts are the possible epicenter of severe dengue in Sabah. J Physiol Anthropol 39:1–11 32795350 10.1186/s40101-020-00230-0 PMC7427916

[8] Kanan M, Naffaa M, Alanazi A, Nasser F, Alsaiari AA, Almehmadi M et al (2024) Genetic variants associated with dengue hemorrhagic fever: a systematic review and meta-analysis. J Infect Public Health 17(4):579–587 38368646 10.1016/j.jiph.2024.02.001

[9] Guerra-Espinosa C, Jiménez-Fernández M, Sánchez-Madrid F, Serrador JM (2024) ICAMs in immunity, intercellular adhesion and communication. Cells 13(4):339 38391953 10.3390/cells13040339 PMC10886500

[10] Yuan H, Jiang M, Fang H, Tian H (2025) Recent advances in poly (amino acids), polypeptides, and their derivatives in drug delivery. Nanoscale 17(7):3549–3584 39745097 10.1039/d4nr04481a

[11] Yang J, Chen X, He X, Fang X, Liu S, Zou L et al (2024) Tanreqing injection demonstrates anti-dengue activity through the regulation of the NF-κB-ICAM-1/VCAM-1 axis. Phytomedicine 130:155764 38797030 10.1016/j.phymed.2024.155764

[12] Nolitriani N, Mariko R, Mayetti M (2021) Soluble vascular cell adhesion molecule-1 levels and severity of dengue hemorrhagic fever in children. Paediatr Indones 61(6):328–335

[13] Sharma S, Singh SK, Kakkar K, Nyari N, Singh D, Dhole TN et al (2016) Association of ICAM-1 K469E polymorphism with dengue infection in North Indian population. Microb Pathog 96:80–84 27179462 10.1016/j.micpath.2016.05.006

[14] Yeh TM, Liu SH, Lin KC, Kuo C, Kuo SY, Huang TY et al (2013) Dengue virus enhances thrombomodulin and ICAM-1 expression through the macrophage migration inhibitory factor induction of the MAPK and PI3K signaling pathways. PLoS One 8(1):e55018 23383040 10.1371/journal.pone.0055018 PMC3557271

[15] Lin CF, Chiu SC, Hsiao YL, Wan SW, Lei HY, Shiau AL et al (2005) Expression of cytokine, chemokine, and adhesion molecules during endothelial cell activation induced by antibodies against dengue virus nonstructural protein 1. J Immunol 174(1):395–403 15611263 10.4049/jimmunol.174.1.395

[16] Vitoria WO, Thomé LS, Kanashiro-Galo L, Carvalho LVD, Penny R, Santos WLC et al (2019) Upregulation of intercellular adhesion molecule-1 and vascular cell adhesion molecule-1 in renal tissue in severe dengue in humans: effects on endothelial activation/dysfunction. Rev Soc Bras Med Trop 52:e20180353 31778418 10.1590/0037-8682-0353-2018

[17] Gauderman WJ (2006) QUANTO 1.2: A computer program for power and sample size calculations for genetic-epidemiology studies. Los Angeles: University of Southern California; Available from: http://biostats.usc.edu/Quanto.html

[18] Fluidigm Corporation (2011) SNP Type™ genotyping system: user guide. Fluidigm Corporation, South San Francisco, CA

[19] Chuang YC, Lei HY, Liu HS, Lin YS, Fu TF, Yeh TM (2011) Macrophage migration inhibitory factor induced by dengue virus infection increases vascular permeability. Cytokine 54(2):222–231 21320786 10.1016/j.cyto.2011.01.013

[20] Popiolek I, Stygar D, Wizner B, Sanak M, Sydor W, Winiarski M et al (2025) Changes of serum cell adhesion molecules as potential markers of inflammation severity, metabolic status and mortality in patients infected with Covid-19. J Physiol Pharmacol. ;76(3) 10.26402/jpp.2025.3.06 40698788

[21] Takahashi T, Iwasaki A (2021) Sex differences in immune responses. Science 371(6527):347–348 33479140 10.1126/science.abe7199

[22] Chakraborty B, Byemerwa J, Krebs T, Lim F, Chang CY, McDonnell DP (2023) Estrogen receptor signaling in the immune system. Endocr Rev 44(1):117–141 35709009 10.1210/endrev/bnac017

[23] Okeke C, Okonkwo R, Ibeh N, Chukwuma O, Okeke C (2023) Assessment of gender differences in some inflammatory cytokines of tuberculosis patients before and during treatment. Afr Health Sci 23(3):336–342 38357187 10.4314/ahs.v23i3.40 PMC10862618

[24] Ng TC, Teo CH, Toh JY, Dunn AG, Ng CJ, Ang TF et al (2022) Factors influencing healthcare seeking in patients with dengue: systematic review. Trop Med Int Health 27(1):13–27 34655508 10.1111/tmi.13695

[25] Ahmed MT, Amin MA (2024) Perilous resurgence of dengue fever in Bangladesh: gender-based perspectives on risk perception and adaptation strategies. Univ J Public Health 11(5):751–760

[26] Padhi S, Sahu S, Pati A, Mohanty AK, Panda AK (2021) Minor allele of intercellular adhesion molecule-1 polymorphism (rs5498 1462A > G) is associated with SARS-CoV-2 infection and related mortality. J Infect Dis 224(4):734–735 34023882 10.1093/infdis/jiab279 PMC8244367

[27] Qidwai T (2021) Intercellular adhesion molecule-1 polymorphisms. Exploration of host genetic factors associated with malaria. Springer, Singapore. p.135 – 45.

